# Bioimpedance Body Measures and Serum Lipid Levels in Masculine Depression

**DOI:** 10.3389/fpsyt.2022.794351

**Published:** 2022-07-19

**Authors:** Claudia von Zimmermann, Lena Brückner, Christiane Mühle, Christian Weinland, Johannes Kornhuber, Bernd Lenz

**Affiliations:** ^1^Department of Psychiatry and Psychotherapy, Friedrich-Alexander University Erlangen-Nürnberg (FAU), Erlangen, Germany; ^2^Department of Addictive Behavior and Addiction Medicine, Central Institute of Mental Health (CIMH), Medical Faculty Mannheim, Heidelberg University, Mannheim, Germany

**Keywords:** serum lipids, cholesterol, body fat, masculine depression, suicidal behavior

## Abstract

**Background:**

Major depressive disorder (MDD) is a main reason for suicide, and serum lipids are involved in both affective disorders and related suicidal behavior. Moreover, masculine depression has been suggested as a subtype of depression with an increased risk for suicide. Here, we studied the relationship between body measures, serum lipids, suicidal thoughts, and masculine depression.

**Methods:**

Depressed patients (44% women) were divided by a sex-separated median-split into a group of 81 “patients with masculine depression” (mean age ± standard error: 36.4 ± 1.6 years) and a group of 82 “patients with non-masculine depression” (age 45.7 ± 1.6 years) according to the Male Depression Risk Scale. We compared body measures, serum lipid levels, and past suicidal ideation between these groups and explored differences between these groups and 176 healthy controls (51% women; age 37.2 ± 1.0 years).

**Results:**

Patients with masculine depression did not significantly differ from patients with non-masculine depression in any of the body measures, lipid markers, or suicidal thoughts. Compared to healthy controls, both patient groups showed significantly higher body fat (B_[masculine depression]_ = 0.041 and B_[non–masculine depression]_ = 0.050), lower high-density lipoprotein (HDL) cholesterol (B = –0.045 and –0.044), and a higher risk for suicidal thoughts (B = 3.927 and 2.663) than healthy controls. Suicidal thoughts were significantly associated with lower low-density lipoprotein (LDL)/HDL ratios (B = –0.455) in patients with depression and with higher LDL cholesterol levels (B = 0.020) in healthy controls subjects.

**Limitation:**

Correlational study design and focus on in-patients.

**Conclusion:**

In the studied cohort, masculine depression was not significantly associated with the analyzed parameters of body measures, serum lipids, or suicidal thoughts in in-patients with depression.

## Introduction

Major depressive disorder (MDD) is an important public health issue with a high life-time prevalence, responsible for a reduced life expectancy (1, 2). In addition to comorbidities, such as cardiovascular disease and diabetes (1), an increased risk for suicide also contributes to reduced life expectancy.

In countries with high income, women are affected two times more often by depressive episodes, whereas men complete three to four times more often suicide (3, 4). Since suicide risk is strongly increased in subjects with mental disorders like depressive episodes or substance abuse (5), an early diagnosis of such diseases amongst men might be helpful for prevention. Among other reasons, the higher portion of women in patients with depression is assumed to be an artifact of how depression is diagnosed. Thus, a specific subtype of depression with atypical symptoms, the so-called “male depression” has been assumed. Instead of displaying a depressed mood or sadness, this depression type is characterized by more externalizing symptoms, such as anger, aggression, distraction, avoidance, emotional suppression, irritability, substance use, and risk-seeking behavior (6). Moreover, men show lower levels of help-seeking behavior (7, 8), for which reasons could be seen in social norms of traditional masculinity (7, 9, 10), as well as in biological factors, including androgens (5, 11, 12). Thus, tailored approaches are needed for this subtype of depression (9, 13). Contrary to what the name suggests, also women can show this depression subtype (14, 15). Hence, we here use the term masculine depression.

Screening tools like the Gotland Male Depression Scale (GMDS) have been developed for better recognition of affected individuals (16). The GMDS shows a significant overlap with other instruments assessing typical depressiveness, such as the Beck’s Depression Inventory-II (BDI-II). Emphasizing externalizing symptoms, the 22-item Male Depression Risk Scale (MDRS-22) is more appropriate to specifically assess masculine depression (6). An overlap of Cluster B personality has recently been shown (15). Those personality traits are characterized by impulsive and violent-aggressive features, and they are associated with a higher risk for a completed suicide (17, 18).

Low cholesterol has been assumed as a risk factor for suicidal behavior (19, 20). Thus, the administration of cholesterol-lowering drugs was suspected to increase the risk of death by suicide (20). Accordingly, Rabe-Jabłoñska and Poprawska (21) found low levels of total cholesterol and low-density lipoprotein (LDL) cholesterol in patients with depression and acute suicidality. Li et al. (22) were able to show in a huge meta-analysis comprising 7,068 participants that lower concentrations of triglycerides and LDL cholesterol were associated with attempted suicide in depressed patients. Knowles et al. (19) detected a genetic overlap between cholesterol and suicide risk by bivariate polygenic and coefficient-of-relatedness analysis, followed by mediation analysis.

In our former work, we found higher levels of LDL cholesterol in patients with a current major depressive episode (without acute suicidal ideation) than in healthy control subjects (23). Accordingly, the antidepressant effect of adjunctive treatment with statins is well-known (24). Enlarged visceral adipose tissue is associated with dyslipidemia [characterized by hypertriglyceridemia, high LDL, and reduced high-density lipoprotein (HDL) cholesterol levels] (25) and common depression symptoms (26). Sedentary behavior and elevated triglycerides are associated with higher depression risk (27). MDD, especially the atypical subtype, is a strong predictor of obesity and weight gain in the future (28), and obesity increases the risk of future depression (29) and *vice versa*. There is huge clinical evidence supporting the conclusion that depression and obesity can interact with each other in a bidirectional longitudinal association (30). Therefore, we would expect elevated body fat in depressed patients, at least in the group with non-masculine depression.

In this study, we investigated whether body measures, serum lipids, and the likelihood of suicidal thoughts differ between patients with masculine and those with non-masculine depression. For this purpose, we recruited a sex-balanced cohort of depressed patients [according to the International Classification of Diseases (ICD)-10 diagnostic criteria (31)] and applied a median split to subclassify patients with masculine and non-masculine depression. Subsequently, we compared these groups of depressed patients with healthy control subjects.

## Materials and Methods

### Sample Population

The data for this publication were obtained from the Masculine Depression project (15). The study was registered in the German Clinical Trials Register (ID DRKS00015291). We used a prospective, open-label, comparative cohort study design with a single point of data collection. A total of 658 potential participants were screened for this project during a period from May 2017 to November 2019, of which 170 patients and 176 healthy control subjects were included. All study participants were at least 18 years of age, had a body mass index (BMI) <35.0 kg/m^2^, and gave written informed consent to participate in the study.

Recruitment of the patient group and data collection took place by a well-trained medical team at two different research sites in Erlangen, Germany: The Department of Psychiatry and Psychotherapy of the Friedrich-Alexander University Erlangen-Nürnberg (FAU) and the Clinic for Psychiatry, Psychotherapy, Psychosomatics of the Klinikum am Europakanal. The prerequisite for inclusion in the patient group was an in-patient stay at one of the two research sites due to a moderate to severe depressive episode according to the diagnostic criteria of the ICD-10. Study inclusion had to occur within the first 5 days of admission. Present medication or treatment was not considered. Exclusion criteria were psychotic disorders.

The control group participants were recruited *via* flyers and posters in the areas of Nuremberg, Fürth, and Erlangen (Germany), as well as through advertising on social networks. Active recruitment of individuals, who had already participated in studies of the same research institution at earlier points in time, was also carried out. Interested parties were screened by telephone with regard to inclusion and exclusion criteria. Participants with regular intake of psychotropic drugs, a current psychiatric diagnosis according to ICD-10 (except for nicotine dependence), or a history of in-patient treatment were excluded. Control subjects received an allowance of 30 € for participation.

The study visit with data collection was composed of several parts and took a total of approximately 4 h. Healthy control subjects were screened again on the day of inclusion for the presence of a depressive episode according to ICD-10 to avoid incorrect group assignment. This was followed by the collection of body measurements. Subsequently, all participants completed a selection of questionnaires and psychiatric tests, including GMDS, MDRS-22, and suicidal thoughts on the computer under supervision. Blood sampling and an attention-dependent part of the questionnaire had to be finished before 10 a.m. to avoid bias by circadian variation.

### Phenotyping of Depression Symptoms

We used the MDRS-22 and the BDI-II to characterize the present depression more precisely. The MDRS-22 is a multidimensional rating scale specifically designed for detecting externalizing depressive symptoms, as they are more prevalent in masculine depression. It consists of 22 items ranked on an eight-point Likert scale ranging from 0 (“never”) to 7 (“almost always”). It assesses nine symptom clusters within the preceding month which include anger, aggression, distraction from personal problems and avoidance behavior, active suppression of negative emotions, hostility and isolation, irritability, substance abuse, risk-taking, and somatic symptoms (32). The BDI-II is a well-established 21-item questionnaire that assesses the severity of a depressive episode. Each item (e.g., sadness, pessimism) is rated on a four-point Likert scale from 0 to 3 depending on how often the item applied to the emotional experience within the past 2 weeks. The overall score is between 0 and 63, with higher scores indicating more severe depressive symptoms (33). To assess suicidal thoughts, the participants were asked whether they have ever specifically thought about taking their own life.

### Body Measurement

The data collection of the body height of the participants was based on self-report. Body weight (kg), body fat (%), body muscles (%), resting metabolism (kcal), and visceral fat (%) were determined using bioelectrical impedance analysis scales (OMRON). Standard measurements were taken and were performed barefoot with clothing. The body mass index (BMI) (kg/m^2^) was calculated automatically by the bioelectrical impedance analysis scale after entering the body height.

### Blood Analysis

For venous blood samples from the participants, fasting was no requirement. Analysis of the serum lipid parameters triglycerides, total cholesterol, HDL-cholesterol, and LDL-cholesterol was performed by the Central Laboratory of the University Hospital Erlangen, Germany (DIN EN ISO 15189 accredited) by enzymatic photometric assays. The LDL/HDL ratio was calculated.

### Statistical Analyses

The data were analyzed using SPSS for Windows 27.0 (SPSS Inc., Chicago, IL, United States). We excluded seven patients with missing data for the MDRS-22. Student’s *t*-tests were employed to test for differences in two independent groups; we used Levene’s test for homogeneity of variance, and the statistics were adjusted where necessary. Differences in frequencies were tested using χ^2^ tests.

The sample of depressed patients was divided into a group of 81 “patients with masculine depression” and 82 “patients with non-masculine depression” according to sex-separated median values of an adapted MDRS-22 (6) which was based on a 5-point scale (15). For reasons of comparability with other studies, we here report transformed values to fit the original 8-point scale ranging from “not at all” (score 0) to “almost always” (score 7). Mean values of items were calculated for the sub-scales and the sum scale.

We used binary logistic regression models with masculine depression vs. non-masculine depression as a primary dependent variable and masculine depression vs. healthy controls and non-masculine depression vs. healthy controls as further dependent variables. Body measures, serum lipid levels, suicidal thoughts together with sex, the BDI-II score (to account for differences in depression severity in the models comparing the masculine depression group with the non-masculine depression group), and age were used as predictors. Moreover, we employed binary regression models separately for depressed patients and healthy controls with suicidal thoughts as dependent variable and body measure or serum lipid parameter together with sex, BDI-II score, and age as predictors. We report means, standard errors of the mean, and regression coefficients B. The binary regression models were validated using bias-corrected and accelerated bootstrap (1,000 resamples). A *p*-value < 0.05 was considered significant.

## Results

### Cohort Characteristics

Tables 1, 2 show cohort characteristics and comparisons of the two patient groups and the group of healthy control subjects. Relative to patients with non-masculine depression, patients with masculine depression had a significantly higher likelihood to be smokers (47 vs. 30%), to be unmarried (72 vs. 52%), and to be younger (36.4 vs. 45.7 years), and showed a higher depression severity according to the BDI-II score (37.3 vs. 28.7).

**TABLE 1:** Cohort characteristics.

	Patients with masculine depression (N = 81)			Patients with non-masculine depression (N = 82)			Healthy control subjects (N = 176)			Patients with masculine depression vs. patients with non-masculine depression		Patient with masculine depression vs. controls		Patients with non-masculine depression vs. controls	
						
	N	M/F	SEM	N	M/F	SEM	N	M/F	SEM	χ^2^ or t	*p*	χ^2^ or t	*p*	χ^2^ or t	*p*
% Women	81	44		82	44		176	51		0.0	0.944	1.0	0.319	1.2	0.279
% Smoker	81	47		81	30		175	6		5.1	**0.024**	61.7	**<0.001**	27.5	**<0.001**
% Living in a current partnership	79	48		77	55		176	68		0.6	0.421	9.3	**0.002**	4.3	**0.038**
% Married	79	28		80	48		175	27		6.5	**0.011**	0.0	0.945	9.9	**0.002**
% Divorced	79	14		78	21		176	14		1.2	0.274	0.0	0.953	1.6	0.207
Age (years)	81	36.4	1.6	82	45.7	1.6	176	37.2	1.0	–4.1	**<0.001**	–0.4	0.684	4.5	**<0.001**
BDI-II score	80	37.3	1.2	81	28.7	1.1	174	3.4	0.3	5.2	**<0.001**	27.9	**<0.001**	21.5	**<0.001**
MDRS-22 score	81	2.6	0.1	82	1.1	0.0	176	0.4	0.0	15.2	**<0.001**	24.2	**<0.001**	14.5	**<0.001**

*The table shows the valid number of subjects analyzed (N), means (M) or relative frequencies (F), standard errors of the mean (SEM), and the results of χ^2^ and Student’s t-tests. BDI- II, Beck Depression Inventory-II; MDRS-22, Male Depression Risk Scale 22 items. p < 0.05 in bold.*

**TABLE 2:** Descriptive characteristics of body measures, serum lipids, and suicidal behavior.

	Patients with masculine depression (N = 81)			Patients with non-masculine depression (N = 82)			Healthy control subjects (N = 176)		
	N	M/F	SEM	N	M/F	SEM	N	M/F	SEM
BMI (kg/m^2^)	81	25.8	0.5	82	26.3	0.4	175	25.0	0.3
Body fat (%)	78	28.9	1.1	81	29.6	1.0	175	27.7	0.6
Body muscles (%)	77	32.4	0.7	81	31.4	0.6	175	32.6	0.4
Resting metabolism (kcal)	78	1652.6	28.5	81	1629.1	27.8	175	1598.5	19.8
Visceral fat (%)	78	7.6	0.4	81	8.9	0.5	175	7.0	0.3
Triglycerides (mg/dl)	81	135.0	10.0	82	139.0	8.9	176	116.9	5.7
Total cholesterol (mg/dl)	81	202.8	5.6	82	210.3	5.9	176	201.3	3.2
HDL cholesterol (mg/dl)	81	51.1	1.6	82	52.1	1.6	176	58.0	1.0
LDL cholesterol (mg/dl)	81	131.6	4.3	82	136.4	4.6	176	125.6	2.5
LDL/HDL ratio	81	2.6	0.2	82	2.6	0.2	176	2.3	0.1
% With suicidal thoughts	71	78.9		72	50.0		169	7.7	

*The table shows the valid number of subjects analyzed (N), means (M) or relative frequencies (F), and standard errors of the mean (SEM). BMI, body mass index; HDL cholesterol, high-density lipoprotein cholesterol; LDL cholesterol, low-density lipoprotein cholesterol.*

### Patients With Masculine Depression vs. Patients With Non-masculine Depression

In models taking into account sex, BDI-II scores, and age, the group of patients with masculine depression did not significantly differ from the group of patients with non-masculine depression in any of the body measures, lipid markers, or likelihood of suicidal thoughts (Table 3). Female sex was significantly related to the group of patients with non-masculine depression (B = 1.020) in the model that included suicidal thoughts as a predictor. Higher BDI-II scores (B from 0.092 to 0.097) and younger age (B from –0.052 to –0.046) were significantly associated with the patients with masculine depression (Table 3).

**TABLE 3:** Binary logistic regression to differentiate between patients with masculine depression and those with non-masculine depression.

		Body measure, serum lipid level, or suicidal thoughts			Sex			BDI-II			Age		
					
	N	B	Wald	*p*	B	Wald	*p*	B	Wald	*p*	B	Wald	*p*
**Patients with Masculine Depression vs. Patients with Non-Masculine Depression**													
BMI (kg/m^2^)	161	0.005	0.0	0.902	0.642	2.7	0.100	0.092	21.9	<0.001*	–0.051	14.6	<0.001*
Body fat (%)	158	–0.005	0.0	0.860	0.556	1.3	0.251	0.095	22.4	<0.001*	–0.047	12.8	<0.001*
Body muscles (%)	157	0.023	0.2	0.648	0.495	0.8	0.383	0.096	22.4	<0.001*	–0.048	12.1	0.001*
Resting metabolism (kcal)	158	0.001	1.6	0.206	0.118	0.0	0.830	0.097	22.7	<0.001*	–0.047	12.9	<0.001*
Visceral fat (%)	158	–0.013	0.1	0.819	0.649	2.3	0.131	0.094	22.3	<0.001*	–0.046	9.6	0.002*
Triglycerides (mg/dl)	161	0.000	0.0	0.896	0.630	2.4	0.125	0.092	21.9	<0.001*	–0.050	15.0	<0.001*
Total cholesterol (mg/dl)	161	0.002	0.2	0.687	0.656	2.8	0.093	0.092	21.9	<0.001*	–0.052	14.4	<0.001*
HDL cholesterol (mg/dl)	161	0.007	0.3	0.608	0.726	3.0	0.084	0.093	22.1	<0.001*	–0.051	15.3	<0.001*
LDL cholesterol (mg/dl)	161	0.001	0.1	0.784	0.643	2.7	0.099	0.092	21.8	<0.001*	–0.051	14.3	<0.001*
LDL/HDL ratio	161	–0.067	0.3	0.561	0.719	3.1	0.079	0.094	22.1	<0.001*	–0.050	14.7	<0.001*
Suicidal thoughts (no vs. yes)	142	0.774	3.2	0.074	1.020	4.9	**0.027**	0.096	18.2	<0.001*	–0.050	11.8	0.001*

The table shows the valid number of subjects analyzed (N) and the results of binary logistic regression analyses. BDI-II, Beck Depression Inventory-II; BMI, body mass index; HDL cholesterol, high-density lipoprotein cholesterol; LDL cholesterol, low-density lipoprotein cholesterol. p < 0.05 in bold, *also significant in bootstrap analysis. Coding: Patients with Non-Masculine Depression = 0 vs. Patients with Masculine Depression = 1; suicidal thoughts no = 0 vs. yes = 1; women = 0 vs. men = 1.

### Patients With Masculine Depression vs. Healthy Control Subjects

In the statistical models with sex and age as predictors, the patients with masculine depression showed significantly higher body fat (B = 0.041), lower HDL cholesterol (B = –0.045), and a higher likelihood for suicidal thoughts (B = 3.927) than the healthy control subjects (Table 4A). Female sex was significantly related to the control group in models, including body fat (B = 0.721) and suicidal thoughts (B = 0.884). Patients with masculine depression did not significantly differ from healthy control subjects in terms of age (Table 4A).

**TABLE 4:** Binary logistic regression to differentiate between patients (both with masculine depression and those with non-masculine depression) and healthy controls.

		Body measure, serum lipid level, or suicidal thoughts			Sex			Age		
				
	N	B	Wald	*p*	B	Wald	*p*	B	Wald	*p*
**(A) Patients with Masculine Depression vs. Healthy Controls**										
BMI (kg/m^2^)	256	0.057	2.3	0.125	0.182	0.4	0.515	–0.007	0.5	0.467
Body fat (%)	253	0.041	4.1	0.042*	0.721	4.0	0.045*	–0.004	0.2	0.699
Body muscles (%)	252	–0.062	2.7	0.098	0.832	3.8	0.052	–0.008	0.6	0.429
Resting metabolism (kcal)	253	0.001	1.6	0.199	–0.208	0.2	0.649	–0.001	0.0	0.890
Visceral fat (%)	253	0.056	1.4	0.229	0.072	0.1	0.821	–0.008	0.5	0.474
Triglycerides (mg/dl)	257	0.002	2.0	0.162	0.121	0.2	0.678	–0.006	0.3	0.572
Total cholesterol (mg/dl)	257	0.001	0.1	0.700	0.262	0.9	0.335	–0.006	0.3	0.571
HDL cholesterol (mg/dl)	257	–0.045	12.2	<0.001*	–0.105	0.1	0.723	0.007	0.4	0.503
LDL cholesterol (mg/dl)	257	0.006	1.9	0.167	0.199	0.5	0.470	–0.010	0.9	0.345
LDL/HDL ratio	257	0.308	3.6	0.057	0.069	0.1	0.813	–0.006	0.4	0.540
Suicidal thoughts (no vs. yes)	240	3.927	81.8	<0.001*	0.884	4.1	0.042*	–0.004	0.1	0.791
**(B) Patients with Non-Masculine Depression vs. Healthy Controls**										
BMI (kg/m^2^)	257	0.057	2.0	0.159	0.068	0.1	0.816	0.038	13.9	<0.001*
Body fat (%)	256	0.050	5.7	0.017*	0.762	4.2	**0.041**	0.037	13.3	<0.001*
Body muscles (%)	256	–0.100	6.1	0.013*	1.013	5.4	0.020*	0.032	9.5	0.002*
Resting metabolism (kcal)	256	0.000	0.0	0.963	0.222	0.2	0.650	0.040	16.3	<0.001*
Visceral fat (%)	256	0.072	2.3	0.127	–0.071	0.0	0.833	0.031	7.4	0.007*
Triglycerides (mg/dl)	258	0.002	1.3	0.249	0.051	0.0	0.864	0.040	15.8	<0.001*
Total cholesterol (mg/dl)	258	–0.001	0.1	0.786	0.166	0.3	0.554	0.042	15.7	<0.001*
HDL cholesterol (mg/dl)	258	–0.044	13.1	<0.001*	–0.323	1.0	0.310	0.050	22.3	<0.001*
LDL cholesterol (mg/dl)	258	0.003	0.4	0.534	0.152	0.3	0.589	0.039	14.0	<0.001*
LDL/HDL ratio	258	0.233	2.1	0.151	0.011	0.0	0.970	0.038	14.4	<0.001*
Suicidal thoughts (no vs. yes)	241	2.663	43.3	<0.001*	0.478	1.9	0.166	0.041	12.0	0.001*

The table shows the valid number of subjects analyzed (N) and the results of binary logistic regression analyses. BMI, body mass index; HDL cholesterol, high-density lipoprotein cholesterol; LDL cholesterol, low-density lipoprotein cholesterol. p < 0.05 in bold, *also significant in bootstrap analysis. Coding: (A) Healthy Controls = 0 vs. Patients with Masculine Depression = 1; (B) Healthy Controls = 0 vs. Patients with Non-Masculine Depression = 1; suicidal thoughts no = 0 vs. yes = 1; women = 0 vs. men = 1.

### Patients With Non-masculine Depression vs. Healthy Control Subjects

In the statistical models, including sex and age as predictors, we found significantly higher body fat (B = 0.050), lower body muscles (B = –0.100), lower HDL cholesterol (B = –0.044), and a higher likelihood for suicidal thoughts (B = 2.663) in patients with non-masculine depression than in healthy control subjects (Table 4B). Female sex was significantly linked to the control group in models that included body fat (B = 0.762) and body muscles (B = 1.013), and younger age was significantly related to the healthy control group in all models (B from 0.031 to 0.050) (Table 4B).

### Body Measures and Lipid Markers in Suicidal Thoughts

Suicidal thoughts were significantly associated with lower LDL/HDL ratios (B = –0.455) in patients with depression and with higher LDL cholesterol levels (B = 0.020) in healthy control subjects. For healthy controls, female sex was significantly related to a higher likelihood for suicidal thoughts than male sex in all models of lipid markers (B from –1.923 to –1.492). In all models of patients with depression except for visceral fat, higher BDI-II score (B from 0.063 to 0.068) and younger age (B from –0.036 to –0.027) were related to a higher likelihood for suicidal thoughts (Table 5).

**TABLE 5:** Binary logistic regression to predict suicidal thoughts by body measures and serum lipids.

		Body measure/Serum lipid parameter			Sex			BDI-II			Age		
	N	B	Wald	*p*	B	Wald	*p*	B	Wald	*p*	B	Wald	*p*
**A Depressed Patients**													
BMI (kg/m^2^)	142	–0.016	0.1	0.722	0.459	1.2	0.266	0.063	11.8	0.001*	–0.033	6.2	0.013*
Body fat (%)	139	–0.016	0.3	0.569	0.227	0.2	0.667	0.065	12.0	0.001*	–0.030	5.2	0.023*
Body muscles (%)	139	0.015	0.1	0.783	0.286	0.2	0.645	0.065	11.9	0.001*	–0.030	4.5	0.034*
Resting metabolism (kcal)	139	0.000	0.0	0.911	0.372	0.4	0.510	0.065	12.0	0.001*	–0.031	5.5	0.019*
Visceral fat (%)	139	–0.028	0.2	0.624	0.510	1.3	0.263	0.065	12.0	0.001*	–0.028	3.5	0.061
Triglycerides (mg/dl)	142	–0.002	0.8	0.369	0.569	1.7	0.192	0.064	12.0	0.001*	–0.032	6.1	0.013*
Total cholesterol (mg/dl)	142	–0.006	2.1	0.148	0.413	1.0	0.318	0.064	11.9	0.001*	–0.027	4.0	0.044*
HDL cholesterol (mg/dl)	142	0.017	1.2	0.282	0.606	1.9	0.168	0.065	12.3	<0.001*	–0.036	7.4	0.006*
LDL cholesterol (mg/dl)	142	–0.010	3.5	0.063	0.473	1.3	0.255	0.065	12.4	<0.001*	–0.027	3.9	0.049*
LDL/HDL ratio	142	–0.455	5.0	0.025*	0.740	2.9	0.091	0.068	13.2	<0.001*	–0.030	5.0	0.026*
**B Healthy Controls**													
BMI (kg/m^2^)	166	–0.036	0.1	0.714	–1.258	3.1	0.077	0.057	0.6	0.439	0.020	0.8	0.377
Body fat (%)	166	0.022	0.3	0.608	–1.092	1.7	0.190	0.043	0.4	0.547	0.016	0.5	0.465
Body muscles (%)	166	–0.111	1.8	0.180	–0.421	0.2	0.663	0.038	0.3	0.588	0.011	0.3	0.608
Resting metabolism (kcal)	166	0.000	0.0	0.971	–1.370	1.3	0.260	0.050	0.5	0.478	0.017	0.6	0.423
Visceral fat (%)	166	0.005	0.0	0.968	–1.352	2.6	0.104	0.050	0.5	0.485	0.017	0.4	0.522
Triglycerides (mg/dl)	167	0.003	0.5	0.491	–1.533	4.1	0.043*	0.045	0.4	0.524	0.015	0.5	0.482
Total cholesterol (mg/dl)	167	0.015	3.5	0.062	–1.492	4.6	0.033*	0.027	0.1	0.731	–0.006	0.1	0.821
HDL cholesterol (mg/dl)	167	–0.019	0.5	0.482	–1.537	4.4	0.037*	0.048	0.5	0.497	0.023	1.0	0.322
LDL cholesterol (mg/dl)	167	0.020	4.1	0.042*	–1.656	5.3	0.021*	0.025	0.1	0.750	–0.003	0.0	0.891
LDL/HDL ratio	167	0.783	3.6	0.058	–1.923	6.1	0.013*	0.029	0.2	0.691	0.013	0.4	0.549

The table shows the valid number of subjects analyzed (N) and the results of binary logistic regression analyses. BDI-II, Beck Depression Inventory-II; BMI, body mass index; HDL cholesterol, high-density lipoprotein cholesterol; LDL cholesterol, low-density lipoprotein cholesterol. p < 0.05 in bold, *also significant in bootstrap analysis. Coding: Suicidal thoughts no = 0 vs. yes = 1; women = 0 vs. men = 1.

## Discussion

This project was conducted to analyze the differences between masculine and non-masculine depression subtypes in terms of body measures, serum lipids, and suicidal behavior. Moreover, we aimed at testing variations between these two depression groups and healthy control subjects.

To our knowledge, this is the first study to show that patients with masculine depression do not significantly differ from patients with non-masculine depression concerning body measures or lipid markers. The risk of suicidal thoughts did also not significantly vary between patients with masculine and those with non-masculine depression in our study cohort, although there was a trend for such an effect. However, patients of both groups, i.e., with masculine and non-masculine depression, showed higher body fat, lower HDL cholesterol, and a higher risk for suicidal thoughts than the healthy controls. The group of patients with non-masculine depression was also found to have a lower percentage of body muscles than healthy control subjects. Also, sex and age were significant predictors in the binary regression models.

The findings of higher body fat in both patient groups (vs. healthy controls) widen the results of our former research, which demonstrated that higher body fat mass and lower body muscle mass correlated significantly with stronger depression severity (34). It also agrees with former studies showing elevated body fat to associate with depressive symptoms (26, 35).

This study observed a lower percentage of body muscles [indicative of less physical activity which is typical for depressed patients (34)] only in patients with non-masculine depression, but not in those with masculine depression, compared to controls. This might suggest that masculine depression (vs. non-masculine depression) is associated with higher physical activity and therefore normal muscle percentage; however, this hypothesis needs to be validated in future research.

Previous reports agree with this study’s finding of lower HDL cholesterol in depressed patients of both groups with masculine and non-masculine depression relative to healthy control subjects. In an earlier study comparing patients with a current major depressive episode relative to healthy controls, we detected higher levels of LDL cholesterol and LDL/HDL ratio and also lower levels of HDL cholesterol in the patients (although the HDL cholesterol difference did not reach significance) (23). Accordingly, former studies have also shown lower levels of HDL cholesterol in psychiatric populations (36, 37). Melin et al. (38) found lower HDL cholesterol levels to associate with depression in patients with type 1 diabetes. Recent research has focused on immune-metabolic dysregulation in relation to different phenotypes of depression. Lamers et al. (39) were able to show that the atypical energy-related depression symptom dimension is linked to poorer metabolic health, while the melancholic symptom dimension is related to relatively better metabolic health. Patients with those atypical symptoms might be at the highest risk of developing cardio-metabolic comorbidities over time in comparison to patients with other depression pathologies (36). However, we did not find such elevated risk parameters in the patients suffering from masculine depression vs. those with non-masculine depression.

We were able to show a higher prevalence of impulsive, borderline, and dissocial personality dimensions in patients with masculine depression in comparison to patients with non-masculine depression in the same study cohort (15). Those cluster B personality traits have been recognized as risk factors for suicide completion (18). However, it is important to differentiate between suicidal thoughts, suicide attempts, and suicide completion. For suicidal thoughts, we did not find significant differences between patients with masculine depression and those with non-masculine depression. According to the androgen model of suicide completion, there might be different predictors for suicidal thoughts, suicide attempts, and suicide completion, and suicide completion appears to be related to male sex and androgen activities (5).

In our study cohort, suicidal thoughts were significantly associated with lower LDL/HDL ratios in patients with depression. These findings are supported by data suggesting that reduced LDL levels (22) and elevated HDL levels (40) might increase the risk for suicidal behavior in depressed patients. However, former studies also showed lowered levels of HDL cholesterol in depressed persons with suicidal ideation in Asian as well as European populations (36, 41). Another study found in women but not men of a national Asian sample of the general population an association between low HDL cholesterol and increased prevalence of suicide attempts, which might however underlie different mechanisms as compared to suicidal thoughts (5, 42).

Our results were also different from Messaoud et al. (43) who did not find any differences in HDL and LDL cholesterol levels between depressed patients with suicide attempts and without suicide attempts. While Messaoud et al. (43) investigated the relationship between suicidal attempts and lipid levels, our study puts its focus on suicidal thoughts. This important difference might explain the varying results. Future studies are needed to investigate whether masculine depression is able to predict specifically suicide attempts and/or completion.

### Limitations

This study is subject to limitations. We used an associational study design, which does not allow for conclusions regarding causality or directionality. Also, a rather small study population was analyzed. The sample size would have been sufficient to detect effects of at least d = 0.44 in *t*-tests (sample size: 81 patients with masculine depression, 82 patients with non-masculine depression; power (1 – β error probability): 0.8; α error probability: 0.05). It remains to be shown whether the trend for a difference in risk for suicidal thoughts between masculine and non-masculine depression (B = 0.774, *p* = 0.074) reaches significance in a larger group. We did not differentiate according to clinical characteristics, such as episode duration or former episodes of depression. Further, we did not assess medication. Given the metabolic side effect profiles of certain psychotropic medications, this is an important weakness. Another limitation was that we only included hospitalized patients already diagnosed with a moderate to severe depressive episode according to the ICD-10 and thus might have missed patients with masculine depression that would show less of the typical depression symptoms.

A strength of our study is that we considered relevant influencing factors in the statistical models and included sex, BDI-II, and age.

In our separate groups of patients with masculine and non-masculine depression, we were not able to replicate our former results showing significantly higher levels of LDL cholesterol and a higher LDL/HDL ratio in patients with a current major depressive episode than in healthy control subjects (23). However, we found a trend for higher LDL/HDL ratios in patients with masculine depression than in healthy control subjects (B = 0.308, *p* = 0.057) supporting our previous findings.

## Conclusion

In line with former studies, we were able to replicate lower HDL cholesterol levels in depressed patients (with masculine and with non-masculine depression) in comparison to healthy control subjects. We also found suicidal thoughts to relate to lower LDL/HDL ratios in the total group of patients with depression. However, we did not find any significant differences in body measures, serum lipid levels, or risk for suicidal thoughts between patients with masculine and those with non-masculine depression in this in-patients study cohort. Future studies should include depressed out-patients as well as patients suffering from masculine depression, but not fulfilling the criteria of a moderate or severe depressive episode according to ICD-10. Analyzing physical activity shall also give insight into the association between depression, masculine depression, body measures, serum lipid levels, and suicidal thoughts.

## Data Availability Statement

The raw data supporting the conclusions of this article will be made available by the authors, without undue reservation.

## Ethics Statement

The study was reviewed and approved by the Ethics Committee of the Medical Faculty of the Friedrich-Alexander University Erlangen-Nürnberg (FAU) (ID 194_16 B). The participants provided their written informed consent to participate in this study.

## Author Contributions

LB, CM, CW, JK, and BL conceived and designed the experiments. LB, CM, CW, and BL performed the experiments. CZ, LB, and BL analyzed the data and wrote the manuscript. CM, CW, and JK commented on the manuscript and provided intellectual input. All authors contributed to the article and approved the submitted version.

## Conflict of Interest

The authors declare that the research was conducted in the absence of any commercial or financial relationships that could be construed as a potential conflict of interest.

## Publisher’s Note

All claims expressed in this article are solely those of the authors and do not necessarily represent those of their affiliated organizations, or those of the publisher, the editors and the reviewers. Any product that may be evaluated in this article, or claim that may be made by its manufacturer, is not guaranteed or endorsed by the publisher.
